# Resection of the Entire Shaft of the Tibia for Necrosis, with Reproduction of the Bone—Recovery

**Published:** 1887-01-01

**Authors:** 


**Resection of the Entire Shaft of the Tibia for Necrosis, with Reproduction of the Bone — Recovery.**— Dr. W. C. Wile, of Newton, Conn., thus writes in the *Brit. Med. Journal*:

On May 8, 1883, I was consulted by the parents of Willie W., in relation to a running sore on the shin of his left leg. On external examination, I found three openings leading through sinuses, which were found to lead to dead bone. The tibia was much enlarged, and the boy showed a decided limp in his gait. He suffered considerable pain, especially at night, which had been controlled chiefly by anodynes. The boy was twelve years old, with hereditary scrofulous tendencies, and had the history of a fall, striking his shin violently against the iron rail of a railroad crossing, causing, at the time, quite a severe contusion, which was followed by considerable inflammation. Soon afterwards, the bone commenced to enlarge, and had continued to increase in size up to the date of my first visit. The sinuses had opened about a year before, and continued to discharge ever since. The boy's general health was poor, he giving every evidence of the exhausting character of the discharges; and it was quite evident that the injuries he had received three years previously had lit upon an inflammation of the periosteum, which had led to grave destruction of bone tissue. It was also quite evident that, if the impairment of the general health was allowed to go on, the result could not be other than death; and the struggle, apparently, could not be a long one. I advised operative interference—of exactly what character, however, it would be hard for me to say until after an exploratory incision had been made. The family consenting, I put the boy upon a month's preparatory treatment, getting the secretions in perfect order, building up the general health with cod-liver oil, iron tonics and liberal diet — beef peptonoids, and Murdock's liquid food — on which I largely depended to build up the patient.

On May 11th, with the assistance of Dr. J. J. Barry, of South Norwalk, who kindly administered the ether, and Dr. S. T. De La Mater, of Bridgeport, I made an incision along the line of the tibia down to the bone, cutting through the diseased periosteum, and carefully lifting it away. I found the bone to be badly diseased; so much so that, after consultation with the two physicians, it was decided to remove the entire shaft. In its removal, I was particularly careful not to injure the periosteum, peeling it up cautiously, and, as nearly as possible, keeping it intact. After removing the shaft, a considerable quantity of diseased bone, at each end, was removed with the gouge. After this was all removed, I packed the cavity with absorbent cotton moistened with a solution of carbolic acid (one to twenty) The boy rallied nicely from the operation; and, under the influence of the same treatment that was pursued in the preparation of the case, healthy granulations sprang up, and there was soon evidence of the production of new bone. After two months, the shaft was strong enough to allow the fitting of the shoe, to which were attached braces at the sides, with the joints at the ankle, the upper ends of which grasped the limb just below the knee by a steel padded band. By this time the external wound had healed, and the boy was put upon crutches. From this time out the progress was uninterrupted. The tibia is now strong and well formed, and the termination of the case is all that could be desired.

